# The Allometry of Coarse Root Biomass: Log-Transformed Linear Regression or Nonlinear Regression?

**DOI:** 10.1371/journal.pone.0077007

**Published:** 2013-10-08

**Authors:** Jiangshan Lai, Bo Yang, Dunmei Lin, Andrew J. Kerkhoff, Keping Ma

**Affiliations:** 1 State Key Laboratory of Vegetation and Environmental Change, Institute of Botany, Chinese Academy of Sciences, Beijing, China; 2 University of Chinese Academy of Sciences, Beijing, China; 3 Department of Biology and Department of Mathematics & Statistics, Kenyon College, Gambier, Ohio, United States of America; DOE Pacific Northwest National Laboratory, United States of America

## Abstract

Precise estimation of root biomass is important for understanding carbon stocks and dynamics in forests. Traditionally, biomass estimates are based on allometric scaling relationships between stem diameter and coarse root biomass calculated using linear regression (LR) on log-transformed data. Recently, it has been suggested that nonlinear regression (NLR) is a preferable fitting method for scaling relationships. But while this claim has been contested on both theoretical and empirical grounds, and statistical methods have been developed to aid in choosing between the two methods in particular cases, few studies have examined the ramifications of erroneously applying NLR. Here, we use direct measurements of 159 trees belonging to three locally dominant species in east China to compare the LR and NLR models of diameter-root biomass allometry. We then contrast model predictions by estimating stand coarse root biomass based on census data from the nearby 24-ha Gutianshan forest plot and by testing the ability of the models to predict known root biomass values measured on multiple tropical species at the Pasoh Forest Reserve in Malaysia. Based on likelihood estimates for model error distributions, as well as the accuracy of extrapolative predictions, we find that LR on log-transformed data is superior to NLR for fitting diameter-root biomass scaling models. More importantly, inappropriately using NLR leads to grossly inaccurate stand biomass estimates, especially for stands dominated by smaller trees.

## Introduction

Accurate estimates of the belowground carbon stocks of forests are critically important for effectively evaluating how climatic change will influence global carbon dynamics [1]. Coarse roots, comprised of the larger, structural roots that provide support for the aboveground biomass, account for most of total belowground biomass carbon in forest ecosystems [2], [3], [4], [5], [6], [7]. Traditionally, belowground forest carbon stocks are estimated from forest inventories and allometric scaling relationships between the trunk diameter and coarse root biomass of a tree [8], [9], [10]. Because of the difficulty associated with excavating the entire root systems of trees, local, site-based allometric equations are uncommon, and estimates are often extrapolated from compositionally or structurally similar sites [8].

The relationship between tree diameter and biomass is highly conserved, with idealized trees exhibiting a power-law relationship [11]. Usually, the relationship is described using a two-parameter power function to fit allometric relationships between stem diameter and coarse root biomass as [2], [12], [13], [14], [15], [16], [17]:
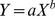
(1)where *Y* is coarse root biomass (kg), *X* usually is diameter at breast height (DBH, cm), *a* and *b* are fitted parameters known as the allometric coefficient and allometric exponent, respectively. Logarithmic transformations are used routinely to fit allometric equations, resulting in a linear model:



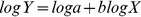
(2)Log-transformation thus simplifies parameter estimation because simple linear regression procedures can be used.

The use of log-transformation has recently been criticized in a variety of applications by Packard and colleagues [18], [19], [20], [21], [22]. Packard *et al.* point out that log-transformed models predict the geometric mean for the response variable instead of arithmetic mean. While arithmetic mean estimates can be obtained from log-transformed models using mathematically simple correction factors [23], Packard et al. claim that log-transformation inherently distorts the relationship between variables and they recommend that allometric analyses should be performed on the arithmetic scale via nonlinear regression.

The choice between linear regression on log-transformed data (hereafter, LR) or nonlinear regression on original data (hereafter, NLR) depends on the distribution of statistical error [24]. NLR fitted to the original data by least squares invokes a statistical model with normally distributed and additive error [24], [25], [26]:
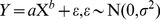
(3)


In contrast, LR fitted to logarithmic transformations of the data by least squares invokes an underlying model with multiplicative log-normally distributed error:
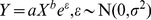
(4)


In cases where the error is approximately multiplicative (lognormal), LR should be used, while NLR should be applied to those data sets with additive normal error [24]. Violation of statistical assumptions of error can lead to biased point estimates as well as inaccurate confidence intervals. Despite its importance in statistical model fitting, error distributions commonly have been omitted from discussions about best practices for fitting allometric equations to biomass data.

Of course, the choice between the two approaches should take the objective of the study into account, as well as the underlying statistical assumptions. One of the most important applications of allometric biomass equations is to convert forest inventory data into stand-level biomass estimates [8]. In general, small values for the response variable have much greater influence on LR parameter estimates, whereas large values for the response variable have much greater influence on NLR, because allometric relationships usually exhibit increasing error variance at larger magnitudes (heteroscedasticity) [21], [22], [27], [28], [29], [30]. As a result, in the context of diameter-biomass allometries, NLR may be a better predictor for the biomass of large trees, while LR is likely to be superior for small trees [21], [22], [31], [32]. In the natural forests, large trees often dominate stand biomass estimates, so NLR should be considered for the estimation of stand-level biomass. Here, we compare LR and NLR models of the allometry between coarse root biomass and diameter and evaluate how the different fitting approaches affect estimates of stand-level belowground biomass.

First, we determine the biomass of coarse root (diameter>2 mm) of 159 trees of three dominant species in a typical subtropical evergreen broad-leaved forest in east China, based on whole-tree excavation. Second, we evaluate the appropriateness of LR and NLR allometric models based on the error distributions in the tree excavation data. Finally, we compare estimates of stand-level belowground biomass from the two allometric models in two different contexts. Locally, we use census data for a nearby 24-ha subtropical forest dynamics plot to contrast LR and NLR estimates in stands dominated by the measured species. Then, to examine how inappropriate models impact biomass estimates when extrapolating to a more remote system, we compare the accuracy of our LR and NLR predictions using known coarse root biomass values for trees from a distant tropical forest.

## Materials and Methods

### Study area

The excavation and forest dynamics studies were conducted in Gutianshan forest area (29°15′N, 118°07′E), Kaihua County, Zhejiang Province in east China. The site is characterized by subtropical monsoon climate, with a mean annual temperature of 15.3°C and the mean annual precipitation of 1964 mm. The substrate consists mainly of granite. The dominant soils can be classified into four types: red soil, red-yellow soil, yellow-red soil and marsh soil. The dominant vegetation type in the Gutianshan is subtropical evergreen broad-leaved forest dominated by *Castanopsis* spp.

No specific permits were required for the described field studies in and outside of Gutianshan forest area. The area is owned and managed by the state and its government and the location including the site for our sampling are not privately-owned or protected in any way. The field studies did not involve any endangered or protected species in this area.

### Data collection

We selected 159 sample trees from three dominant species (*Castanopsis eyrei*, *Schima superba, Pinus massoniana*,) with DBH ranging from 1.1 to 56.5 cm (Table 1 and Appendix S1) and excavated their root systems in the study area in 2008. The DBH of each sampled tree was measured and recorded before being removed by motor chain saw. A back-hoe excavator was then used for whole root system extraction, exercising care to retain lateral roots. They were washed using water and brushes, then weighed after oven-drying at 85°C to constant weight for 7 d or more. The weight of lateral roots that broke off during excavation was approximated using intact lateral roots. The approximation involved measuring the diameter at the point of breakage, removing a randomly selected intact lateral root from the same tree at an equivalent diameter, and adding the weight of this section to the measured weight for the entire root system.

**Table 1:** Characteristics of the 159 sample trees.

Species	No.trees	DBH (cm)	
		Mean	Range
*Castanopsis eyrei*	41	20.2	1.1–40.3
*Schima superba*	60	17.0	1.2–38.3
*Pinus massoniana*	58	20.3	1.3–56.5

### Statistical analysis and model selection

We used likelihood analysis to compare the appropriateness of the two error models (additive and multiplicative error) for each of the three species separately, as well as for 159 sample trees combined, following the method of Xiao *et al.* [24]. For each species and mixed-species data set, we first fit the power-law models using LR and NLR, respectively, to estimate the parameters *a*, *b*, and σ^2^ for each model. We then calculated the likelihood that the data are generated from a normal distribution with additive error:
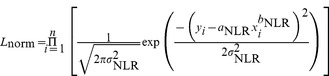
(5)and the likelihood that the data are generated from a lognormal distribution with multiplicative error,
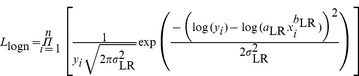
(6)where n is sample size. To select between models, the Akaike information criterion (AICc) for each model was then computed as:
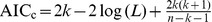
(7)where k is the number of parameters (three in both models) and L is the corresponding likelihood [33]. If AICc-norm –AICc-logn<−2, the assumption of normal error (i.e., NLR) is favored, whereas AICc-norm –AICc-logn>2 indicates that lognormal error (and thus LR) is more reasonable. If |AICc-norm –AICc-logn| ≤2, neither assumption is favored and model averaging is suggested [24].

### Local stand-level belowground biomass estimates

We compared stand level model estimates for LR and NLR using survey data from the Gutianshan forest dynamics plot [34]. In December 2004, a permanent plot covering 24-ha (400×600 m, horizontal distance) was established within the evergreen broad-leaved forest in Gutianshan [35]. The plot was established and data were collected following the plot standards of the Center for Tropical Forest Science network [36]. The first tree census was completed in 2005. All woody stems ≥1 cm DBH were mapped, measured, identified, and tagged. Approximately 140,000 individuals ≥1 cm DBH, belonging to 49 families, 103 genera and 159 species were surveyed, including 26 shrub species, 70 under-story tree species and 63 canopy tree species [35].

Although only three species-specific allometric equations (*Castanopsis eyrei, Schima superba, Pinus massoniana*) were available for our plot, these three species accounted for about 63.3% of the total basal area, and approximately 2/3 of the total aboveground biomass [34]. Thus, if biomass partitioning in forest ecosystem is isometric, as hypothesized [37], [38], these three species should also contribute a similar proportion of the total belowground biomass. The range of diameters present in the study plot encompassed those used to estimate the allometric equations. We used the species-specific models to estimate the belowground biomass for each individual tree of the three dominant species, then used the mixed-species allometric equations to estimate the contribution of each individual tree in all of the remaining species. To make all estimates comparable arithmetic means, we multiplied all of the LR estimates by a correction factor, CF  =  exp(SEE^2^/2), where SEE is the standard error of the estimate [23], [39]. Stand level belowground biomass was then estimated by summation of all individual trees in the plot. To explore how differences between the two models depend on local scale heterogeneity in forest structure, we also subdivided the 24-ha plot into 96, 50 m×50 m (0.25 ha), subplots and conducted separate belowground biomass estimates for each subplot.

### Comparison to remote biomass estimates

Belowground biomass equations are often applied to distant but structurally similar forests because local excavation data are rare and difficult or impossible to obtain. To evaluate the impacts of extrapolating inappropriate models in this way, we compared NLR and LR estimates of belowground biomass to known values for 107 destructively sampled trees from the Pasoh Forest Reserve, Negeri Sembilan, Peninsular Malaysia (2°59′ N, 102° 18′ E) (Data are available from Niiyama et al. 2010 in *Journal of Tropical Ecology* [17]). This primary tropical lowland forest differs substantially from the subtropical Gutianshan forest, though both are evergreen and share similar average annual precipitation levels (Pasoh: 2000 mm/y, Gutianshan: 1964 mm/y). The sampled trees were drawn from 73 species (55 genera) and ranged from 0.4 to 116 cm DBH exceeding the range of the data collected in our study. Using the mixed-species LR and NLR models from the current Gutianshan study, we compared predicted belowground biomass values to the actual, measured values from Pasoh. Since allometric equations are most frequently used to estimate stand biomass, we examined not only the predictions for each individual tree, but also for their sum.

All calculations in this paper were conducted using R statistical language[40].

## Results

**Figure 1:**
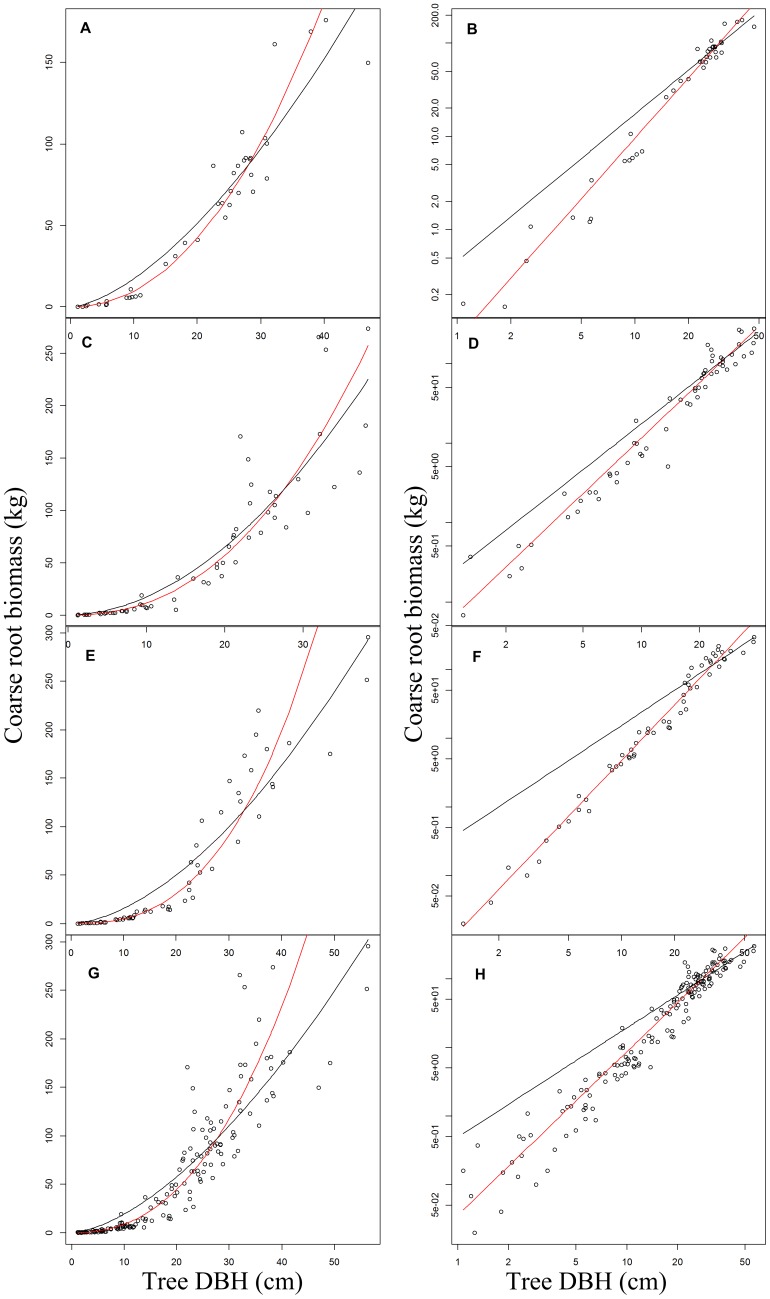
An illustration of NLR (black line) and LR (red line) to fit power-law allometric relationship of diameter-root biomass on both arithmetic (A,C,E.G) and logarithmic scales (B,D,F,H) for three species and mixed species (A,B for *Castanopsis eyrei*, C and D for *Schima superba*, E and F for *Pinus massoniana*, G and H for mixed species).

The assumption of multiplicative lognormal error was strongly supported for each of the three individual species models as well as the mixed-species model. Likelihood analysis of the error structure yielded lower AIC_c_ for the LR models compared to NLR (Table 2); ΔAIC_c_ values (the difference in AIC_c_ between the two models) were large (*C.eyrei = *63.7, *S.superba = *166.2, *P.massoniana = *209.7, mixed-species = 382.8). Residual plots also supported the lognormal assumption (see Appendix S2). Plotted against DBH, residuals for the log-transformed model were more homoscedastic across orders of magnitude in diameter (see Appendix S2).Thus, at least for the Gutianshan data, LR should be favored over NLR to fit diameter-root biomass scaling models. While the fit of the LR and NLR models appear fairly comparable on an arithmetic scale, on a log-scale, the NLR models show a consistent bias, overestimating (often substantially) the coarse root biomass of smaller trees (Fig. 1).This bias resulted from the fact that the allometric exponents (*b* values) estimated by NLR were smaller than those derived from LR, and the NLR allometric coefficients (*a* values) were substantially larger (Table 2).Visualizing the relationship on both arithmetic and log-scales also reinforced the fact that even though *absolute* deviations in coarse root biomass were largest for the large trees, the *proportional* deviations are relatively constant across orders of magnitude in DBH (Fig. 1), which is consistent with the likelihood analysis favoring the lognormal error model. The additive, normal error model yielded unrealistically shallow scaling exponents, because the NLR fit was overly sensitive to the absolute residuals of the largest trees. In contrast, the linear model fitted to log-transformed data (the lognormal error model) is clearly superior for describing coarse root biomass over the full range of diameters for each species individually and for the mixed-species (Fig.1).

**Table 2:** Comparison of two methods for fitting the power-law allometric relationship between stem diameter and coarse roots biomass for three dominant species and mixed species in subtropical evergreen broad-leaved forest in east China.

*Species*	LR				NLR		
	AIC_c_	a (95% CI)	b (95% CI)	CF	AIC_c_	a (95% CI)	b (95% CI)
*Castanopsis eyrei*	285.1	0.064 (0.046, 0.089)	2.15 (2.03, 2.26)	1.06	348.8	0.462 (0.072, 0.852)	1.57 (1.33, 1.82)
*Schima superba*	420.1	0.051 (0.036, 0.070)	2.32 (2.19, 2.44)	1.09	586.3	0.213 (−0.056, 0.482)	1.91 (1.54, 2.28)
*Pinus massoniana*	338.2	0.009(0.007, 0.012)	2.69(2.60,2.79)	1.05	547.9	0.303 (0.054, 0.552)	1.70 (1.48, 1.92)
Mixed species	1147.9	0.031(0.024, 0.040)	2.38 (2.29, 2.48)	1.15	1530.7	0.487 (0.212, 0.763)	1.59 (1.43, 1.75)

**Figure 2:**
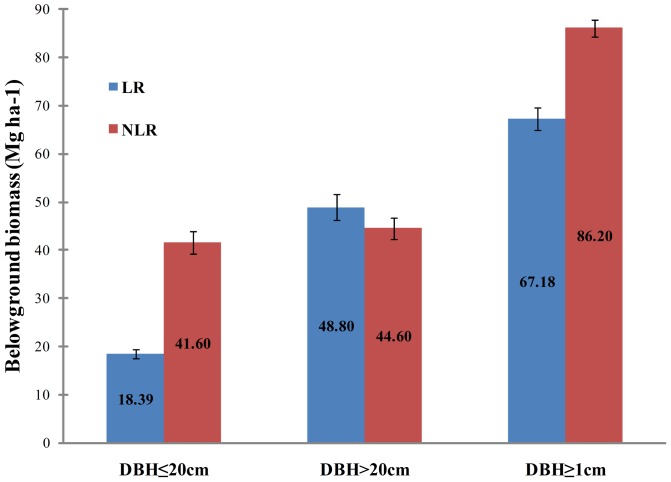
Comparison of estimated belowground biomass based on two types of allometric equations from LR and NLR in Gutianshan 24-ha plot. Error bars show 95% confidence intervals based on 10000 bootstraps over 50×50-m subplots.

**Figure 3:**
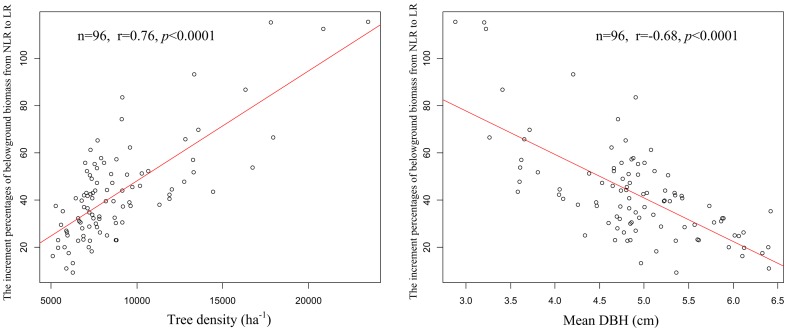
The relationship between estimation bias and tree density and mean DBH over 50×50-m subplots in Gutianshan 24-ha plot. The estimation bias is the percentage overestimate of the NLR coarse root biomass model, compared to the LR model estimate.

When applied to estimate stand-level belowground biomass of the 24-ha Gutianshan plot, the stand-level belowground biomass estimate based on the NLR models was 28% larger than that of the LR models (86.20 ±1.87 Mg ha-1 vs. 67.18±2.27 Mg ha-1, respectively, Fig. 2). The estimates of the two models were similar for trees with DBH >20 cm (48.80±2.84 Mg ha-1for NLR vs. 44.60±2.57 Mg ha-1for LR, Fig. 2), but for smaller individuals (DBH ≤20 cm), NLR estimate were 2.3 times higher than LR (41.60±2.32 Mg ha-1 for NLR vs. 18.39±0.93 for LR, Fig. 2). Across the 96 0.25 ha subplots, we found that the NLR model could overestimate local belowground biomass by up to 116%, with an average overestimate of 43%. Moreover, the magnitude of the bias was positively correlated with tree density (n = 96, r = 0.76,*p*<0.0001) and negatively correlated with mean DBH of subplots (n = 96, r = −0.68, *p*<0.0001, Fig. 3). Thus, the fact that NLR substantially overestimated the coarse root biomass of small trees can strongly bias stand-level belowground biomass estimates, even though large trees contribute a large fraction of stand biomass. These biases result in especially substantial errors in forests dominated by small trees (e.g., young, regenerating stands).

**Figure 4:**
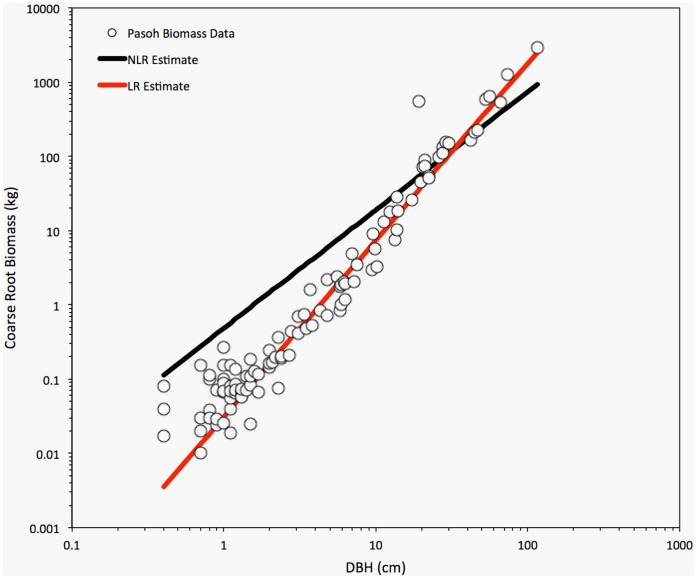
Comparison of LR (red line) and NLR (black line) models parameterized with the Gutianshan data when extrapolated to predict known coarse root biomass values from the Pasoh Forest Reserve (circles).

The same biased overestimate of coarse root biomass occurs when the models are extrapolated to predict the known biomass values from the tropical forest of Pasoh Forest Reserve. The NLR model systematically overestimated the coarse root biomass of small trees, even though many small trees in the Pasoh dataset appear to have relatively large root systems (Fig.4). Interestingly, the NLR model also systematically *underestimated* the coarse root biomass of the largest trees, perhaps because the parameter estimates were overly sensitive to the particular properties of large trees of the Gutianshan data (Fig. 4). As a result, when summed over all of the trees, the NLR model estimate of total coarse root biomass for all 107 trees (4351.6 kg) was 48% lower than the actual measured total (8322.7 kg). In contrast, the LR model estimates fit the Pasoh data remarkably well, given the environmental and taxonomic differences between the two forests (Fig.4), and the total coarse root biomass estimate (7638.6 kg including the correction factor) was within 9% of the actual value. Thus, the bias introduced by erroneously using NLR to parameterize a locally calibrated allometric model will be maintained or exacerbated by extrapolating the model into a new environment.

## Discussion

Our likelihood analysis shows that data on the scaling of coarse root biomass and diameter in trees support a multiplicative, lognormal error model, as is typically found in studies of allometry [41], [42]. Using nonlinear regression with these data on an arithmetic scale yields residuals that are strongly heteroscedastic, exhibiting increasing variation with increasing diameter, as observed in many other studies [8]. Therefore, our results are consistent with previous suggestions that multiplicative log-normal error is the norm for allometric relationships in biology [24], [25], [26], [43], [44] and demonstrates the utility of likelihood methods for selecting the appropriate error model.

The recent spate of publications criticizing log-transformation and recommending NLR [18], [19], [20], [21], [22], [45] are all based on the assumption that the arithmetic scale is somehow more natural and that additive variation should be the default standard for parameter estimation. The use of nonlinear model fitting in biomass estimation is also facilitated by the availability of easy-to-use advanced statistical packages [46], [47], [48], [49]. But as we have shown, NLR parameter estimates are highly sensitive to the largest observations, because large trees display substantially larger absolute variation in root biomass on an arithmetic scale. Log-transformation works for these data precisely because the proportional variation in coarse root biomass is relatively constant across orders of magnitude in tree diameter. With validated statistical methods in place to choose among the different error models, the time for blanket criticism (or defense) of log-transformation is clearly past.

Two ecological factors may in fact compound the bias of NLR root biomass models. First, the development of the root systems of the large trees may be limited by environmental condition and competition [50], [51]. For example, in our study, root biomass shows a tendency to level off for the largest individuals, especially in *Pinus massoniana* (Fig.1F). Since NLR parameter estimates are overly sensitive to large individuals, the resulting exponent is much too shallow. Second, stand level biomass estimates depend not only on the underlying scaling model, but also on stand structure. And although smaller trees may contribute a relatively small proportion of total biomass in forests, the NLR bias will be magnified by the right-skewed size distribution of most forest stands, in which there are many more small saplings than large trees [52], [53], [54], [55]. Thus, as we found here, the NLR models substantially overestimate stand-level biomass in forests that contain many small trees, as would be found early in regeneration or succession. But finding support for these traditional allometric methods has implications well beyond statistical arguments about the mathematical effects of log-transformation and model selection procedures.

Allometric scaling models are one of the key techniques for the estimation of forest carbon stocks, and accurate estimates require careful model development and calibration. If researchers were to follow the advice of Packard et al., and erroneously use NLR-based allometric models, the resulting estimates of the contribution of coarse roots to belowground carbon stock could exhibit systematic errors average 43%, even more than 115%. Easily avoidable biases of this magnitude should concern researchers seeking to estimate local carbon budgets. But more importantly, compounded over national or even heterogeneous continental regions, these errors could bias calculations of both the costs and benefits of climate change mitigation strategies that take into account reforestation and reductions in deforestation, such as REDD+ [56], [57]. These policy concerns are critically important and should not be influenced by misguided statistical debate.

## Supporting Information

Appendix S1 DBH and dry root biomass of the sample trees (n = 159) used for biomass estimation. All sample trees were measured in Gutianshan forest area, Kaihua County, Zhejiang Province in eastern China.
(CSV)

Appendix S2 Analysis of the error structure of the LR models and NLR to fit power-law allometric relationship of diameter-root biomass.
(DOCX)
