# Supplementary material for: The Allometry of Coarse Root Biomass: Log-Transformed Linear Regression or Nonlinear Regression?
Source: PLoS One. 2013 Oct 8;8(10):e77007. doi: 10.1371/journal.pone.0077007 (PMC3792932; doi:10.1371/journal.pone.0077007)
Supplement: Appendix S2 — Analysis of the error structure of the LR models and NLR to fit power-law allometric relationship of diameter-root biomass. (DOCX) [file pone.0077007.s002.docx]

**Appendix S2**: Analysis of the error structure of the LR models and NLR to fit power-law allometric relationship of diameter-root biomass

*Castanopsis eyrei* (n=41)

*Pinus massoniana* (n=58)

*Schima superba* (n=60)

Mixed species (n=159)
